# Association between dietary intake of branched-chain amino acids and sarcopenia and its components: a cross-sectional study

**DOI:** 10.1038/s41598-022-07605-6

**Published:** 2022-04-05

**Authors:** Sara Ebrahimi-Mousavi, Rezvan Hashemi, Amir Bagheri, Ramin Heshmat, Ahmadreza Dorosty-Motlagh, Ahmad Esmaillzadeh

**Affiliations:** 1 Students’ Scientific Research Center, Tehran University of Medical Sciences, Tehran, Iran; 2 Department of Clinical Nutrition, School of Nutritional Sciences and Dietetics, Tehran University of Medical Sciences, Tehran, Iran; 3 Department of Geriatric Medicine, Ziaeian Hospital, Tehran University of Medical Sciences, Tehran, Iran; 4 Department of Community Nutrition, School of Nutritional Sciences and Dietetics, Tehran University of Medical Sciences, Tehran, Iran; 5 Chronic Diseases Research Center (CDRC), Endocrinology and Metabolism Population Sciences Institute, Tehran University of Medical Sciences, Tehran, Iran; 6 Department of Community Nutrition, School of Nutritional Sciences and Dietetics, Tehran University of Medical Sciences, P.O. Box 14155-6117, Tehran, Iran; 7 Obesity and Eating Habits Research Center, Endocrinology and Metabolism Molecular‑Cellular Sciences Institute, Tehran University of Medical Sciences, Tehran, Iran; 8 Department of Community Nutrition, School of Nutrition and Food Science, Isfahan University of Medical Sciences, Isfahan, Iran

**Keywords:** Nutrition, Quality of life

## Abstract

There is no previous study that investigated the association between dietary intake of total and individual branched-chain amino acids (BCAAs) and odds of sarcopenia. The present study aimed to examine the association between dietary intake of BCAAs and sarcopenia and its components among Iranian adults. The data for this cross-sectional study was collected in 2011 among 300 older people (150 men and 150 female) with aged ≥ 55 years. We used a Block-format 117-item food frequency questionnaire (FFQ) to evaluate usual dietary intakes. BCAAs intake was calculated by summing up the amount of valine, leucine and isoleucine intake from all food items in the FFQ. The European Sarcopenia Working Group (EWGSOP) definition was used to determine sarcopenia and its components. Mean age of study participants was 66.8 years and 51% were female. Average intake of BCAAs was 12.8 ± 5.1 g/day. Prevalence of sarcopenia and its components was not significantly different across tertile categories of total and individual BCAAs intake. We found no significant association between total BCAAs intake and odds of sarcopenia (OR for comparison of extreme tertiles 0.48, 95% CI 0.19–1.19, P-trend = 0.10) and its components (For muscle mass 0.83, 95% CI 0.39–1.77, P-trend = 0.63; for hand grip strength 0.81, 95% CI 0.37–1.75, P-trend: 0.59; for gait speed 1.22, 95% CI 0.58–2.57, P-trend = 0.56). After adjusting for potential confounders, this non-significant relationship did not alter. In addition, we did not find any significant association between individual BCAAs intake and odds of sarcopenia or its components. We found no significant association between dietary intakes of BCAAs and sarcopenia in crude model (OR 0.60; 95% CI 0.29–1.26). After controlling for several potential confounders, the result remained insignificant (OR 0.48; 95% CI 0.19–1.19). In this cross-sectional study, no significant association was observed between dietary intakes of total and individual BCAAs and odds of sarcopenia and its components.

## Introduction

Sarcopenia is a geriatric syndrome determined by a progressive and generalized decline in muscle mass, strength and function that associated reduce physical performance, poor quality of life and increased mortality^1–3^. As the global population ages, sarcopenia is becoming a crucial global public health problem^4^. The prevalence of this syndrome is estimated at 1 to 29% in Western countries and 2 to 49% in Asia^5–7^. Sarcopenia imposes significant costs on health care systems, as Janssen et al. estimated that sarcopenia-related annual health care costs in the United States are approximately $ 18 billion^8^.

Various factors have been confirmed to be associated with sarcopenia, including hormonal causes^9^, genetic^10^, physical activity^11^, and nutrients such as protein consumption^12^. In fact, protein affects muscle performance by stimulation and regulation of protein synthesis in muscles, where branched-chain amino acids (BCAAs; leucine, valine, and isoleucine) are metabolized^13–15^. Prior studies using European Working Group on Sarcopenia in Older People (EWGSOP) have shown that sarcopenia is associated with decreased concentrations of BCAAs, leucine and essential amino acids^16, 17^. In addition, supplementation with BCAAs, leucine and/or essential amino acids, resulted in increased protein synthesis in muscle in the elderly^18–21^. However, as far as we know, no previous study has investigated the association between dietary intakes of BCAAs and risk of sarcopenia.

As health conditions improve and life expectancy increases, the population around the world tends to age^22^. The prevalence of sarcopenia is higher in Asian adults than that in westerns^7, 23^. Importantly, the lifestyle and dietary patterns of people in the Middle East are different from those in Western populations^24, 25^. Main dishes in these countries contain high amounts of refined grains, including rice and bread, and dietary sources of protein do not contribute so much to total energy intake^24, 25^. Therefore, dietary intake of BCAAs in this area might be lower than that in western countries. Thus, the present study aimed to investigate the association of BCAAs with sarcopenia and its components among Iranian adults.

## Study participants and methods

### Participants

This cross-sectional study was conducted in Tehran, Iran from May to October 2011. Details of the study design, sampling and data collection process were published previously^26^. A total of 300 elderly people (150 males and 150 females) aged 50 and older were selected by cluster random sampling method in District 6 of Tehran. The heads of each of the 30 clusters were selected according to a 10-digit postal code. In order to ensure the homogeneity of our sample, we did not include individuals with a predisposing cause of sarcopenia with factors other than aging^27^. In other words, people with limited mobility and a history of debilitating diseases such as active cancer, organ failure were not included. Also, people who could not walk without crutches, walkers or assistive devices, or had artificial limbs or prostheses were not included in the study because their lower muscle mass was not comparable to the muscle mass of the general population. The study was conducted in accordance with the Declaration of Helsinki, and the study protocol was approved by the Ethics Committee of the Tehran University of Medical Sciences. All participants gave their written informed consent form before data collection.

### Assessment of dietary intake

We applied a food frequency questionnaire (FFQ) to gather the information about usual dietary intakes of subjects. Participants were requested to report their usual dietary intakes of all foods included in the questionnaire in the preceding year based on daily, weekly or monthly consumption. The questionnaire was a validated one containing 117-food items in Block format. Consumption of foods and nutrients in the preceding year can reflect long-term usual dietary intake, as shown by nutritional epidemiologists^28^. Detailed information on this questionnaire has been reported elsewhere^25, 26^. In addition to the list of food items in the questionnaire, we had included a standard portion size for each item, portion sizes that were generally used by Iranians. Moreover, a frequency response section was also available for food items, in which participants were able to report their consumption of that food item. All the questionnaires were filled in a face-to-face interview by a trained nutritionist. In this interview, the nutritionist was asking the participants to recall and report their consumption of each food item in the questionnaire based on their usual intake in daily, weekly or monthly basis during the last year. Then, all frequencies were changed to daily intake and given the portion sizes of each food item, we converted all the reported foods into grams per day using a booklet of household measures^29^. In order to estimate mean energy and nutrient intakes for each study subject, a modified version of Nutritionist IV software (version 7.0; N-Squared Computing, Salem, OR, USA) was applied^30^. The database of this software had been modified based on the nutrient composition for available Iranian food items. Local foods had also been added to that database.

Dietary intake of BCAAs was calculated by summing up valine, leucine, and isoleucine values in 100 g of each food item for each participant. The main dietary sources contained these amino acids were dairy products, meat, and poultry.

### Assessment of sarcopenia

We used the criteria recommended by the European Working Group in Older People (EWGSOP) to define sarcopenia. In that guideline, sarcopenia has been defined as the existence of both low muscle mass and low muscular strength or low physical performance^27^. In order to calculate muscle mass, first we computed lean mass of the legs and hands (named as Appendicular Skeletal Muscle or ASM) and then through dividing this to height squared (ASM/height^2^), we obtained total muscle mass for each study participant^31^. ASM was evaluated via DEXA scanner (Discovery W S/N 84430). According to the thresholds defined in the EWGSOP, low muscle mass was considered as < 5.45 (kg/m^2^) for women and < 7.26 (kg/m^2^) for men^27^. Muscle strength was assessed by hand grip strength, which was measured by a squeeze bulb dynamometer (c7489-02 Rolyan) calibrated in pound per square inch (psi). The hand grip strength was measured while subjects were sitting in a straight back seat with the shoulders were abducted in the neutral position of the arms and the elbow angle was approximately 90°. Participants in this position had a neutral arm rotation and their wrists deviated from 0° to 30° flexion and 0 to 15 °C. Participants were requested to squeeze the dynamometer as hard as possible for 10 to 30 s. The hand grip strength was assessed for each right and left hand three times with a 30-s rest between measurements. Mean of these three measurements for each hand was considered eventually. Then, by summing up the mean values for both hands, muscle strength was obtained. Low levels of muscle strength was defined according to the age- and gender-specific cut-off points suggested by Merkies et al.^32^. To evaluate the muscle function test, each participant was asked to walk at a normal speed until the end of a 4-m straight course. We recorded the time in seconds with a chronometer^27^. Participants who had gait speeds less than 0.8 m/s were considered as those with low muscle performance^27^.

### Assessment of other variables

Required data on other variables such as demographic characteristics (including age, gender, education and occupation), past medical history and medication use, alcohol intake and smoking status was collected via pretested questionnaires. In order to assess the level of physical activity, the short form of the International Physical Activity Questionnaire (IPAQ) was used, in which the metabolic equivalents-hour per week (MET-h/week) was computed to state the activity level for each study participant. The validity of IPAQ has already been examined in the elderly population^33^. Weight was measured using a digital scale with light clothing. Height was assessed by a wall-mounted tape-meter in standing position without shoes. We measured waist circumference (WC) at the middle of the lower rib margin and iliac crest while people were standing and breathing normally. Finally, body mass index (BMI) was calculated as weight divided by the square of height.

### Statistical analysis

Statistical analyses were conducted using Statistical Package for Social Science (SPSS Inc., Chicago, IL, USA, version 16.0). P values < 0.05 were identified as statistically significant. Initially, the third cut-off points for BCAA, valine, leucine and isoleucine diet were defined in the control group. Then all study participants were classified according to these cut-offs. To compare the general characteristics and dietary intakes across tertiles of BCAAs, we applied one-way ANOVA and chi-square test for continuous and categorical variables, respectively. In order to find the association between BCAAs and odds of sarcopenia and its components, multivariate logistic regression analysis were applied. First, we adjusted for age (continuous), gender (male/female) and total energy intake (kcal/day). In a second model, we applied further adjustment for physical activity (MET-h/week, continues), smoking status (yes/no), alcohol intake (yes/no), medication use (statin, corticosteroid, estrogen, testosterone), and positive history of chronic disease. All models were applied by treating the first tertile of BCAAs as the reference. In order to determine the trend of odds ratios (ORs) across increasing tertiles of BCAAs, the tertiles were considered as an ordinal variable in the logistic regression models.

## Results

In our study population, average intakes of BCAAs, valine, leucine and isoleucine were 12.86 ± 5.18, 4.03 ± 1.65, 5.49 ± 2.25 and 3.33 ± 1.32 g/day, respectively. General characteristics of study participants are shown in Table 1. We found that participants with sarcopenia had a lower BMI than healthy participants. There were no notable differences in other variables between people with and without sarcopenia.

**Table 1:** General characteristics of people with and without sarcopenia.

	Sarcopenia^a^		*P*^‡^
	Yes (n = 54)	No (n = 246)	
Age (year)	68.50 ± 7.99	66.42 ± 7.61	0.07
BMI (kg/m^2^)	24.11 ± 2.59	28.10 ± 4.15	< 0.001
Physical activity (MET-h/w)	19.24 ± 20.47	22.08 ± 24.50	0.42
Female (%)	40.7	53.3	0.09
Alcohol use (%)	13	13.4	0.93
Smoking (%)	14.8	12.2	0.60
**Medical history**			
Diabetes (%)	11	23	0.06
MI (%)	13	12	0.81
CVA (%)	5.6	2.0	0.14
Asthma (%)	1.9	2.0	0.93
Arthritis (%)	1.9	1.6	0.91
**Drug history**			
Sexual hormone use (%)	5.6	2.4	0.22
Statin use (%)	44.4	35.0	0.19
Corticosteroid use (%)	5.6	2.0	0.14

All values are mean ± SD, unless indicated. ^a^Sarcopenia was defined based on European Working Group on Sarcopenia in Older People (EWGSOP) definition^27^. ^‡^ANOVA for continuous variables and Chi-squared test for categorical variables. *BMI* body mass index, *MI* myocardial infarction, *CVA* cerebrovascular accident.

**Figure 1:**
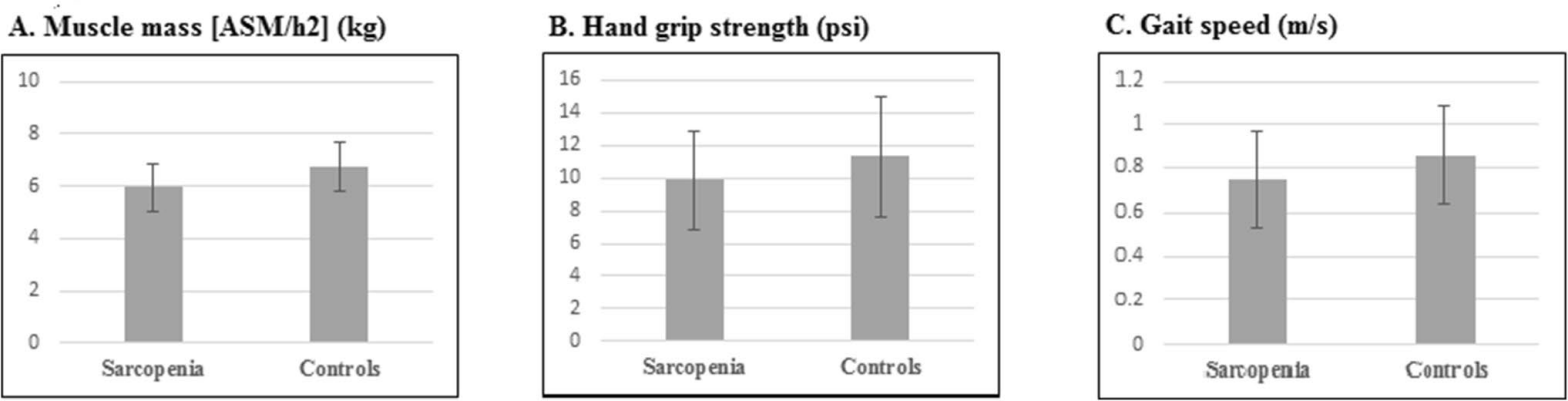
Mean muscle mass, hand grip strength and gait speed between cases and controls. A. Comparison of cases and controls in mean muscle mass, B. Comparison of cases and controls in hand grip strength. C. Comparison of cases and controls in gait speed.

Comparison of cases and controls in terms of components of sarcopenia is shown in Fig. 1. As expected, individuals with sarcopenia had significantly lower mean values of muscle mass (6.75 ± 0.95 vs. 5.95 ± 0.88, P < 0.001, Fig. 1A), hand grip strength (11.30 ± 3.64 vs. 9.85 ± 2.96, P = 0.007, Fig. 1B) and gait speed (0.86 ± 0.22 vs. 0.75 ± 0.22, P = 0.001, Fig. 1B) Fig. 1C, compared with healthy individuals.

Table 2 indicates comparison of dietary nutrients intakes between sarcopenic and non-sarcopenic subjects. There were no significant differences in terms of energy, total BCAAs, leucine, isoleucine, valine, proteins, carbohydrates, proteins, fats, thiamin, riboflavin, niacin, pantothenic acid, pyridoxin, folate, B12, biotin, iron, calcium, magnesium, zinc and tryptophan between the two groups.

**Table 2:** Comparison of dietary intakes of participants with and without sarcopenia.

	Sarcopenia^a^		*P*^‡^
	Yes (n = 54)	No (n = 246)	
Energy (kcal/day)	2323 ± 1331	2249 ± 819	0.59
Total BCAAs (g/day)	12.78 ± 5.70	12.88 ± 5.08	0.90
Leucine (g/day)	5.48 ± 2.48	5.50 ± 2.20	0.95
Isoleucine (g/day)	3.30 ± 1.50	3.33 ± 1.27	0.86
Valine (g/day)	3.99 ± 1.72	4.04 ± 1.64	0.85
Protein (g/day)	85.5 ± 39.38	86.18 ± 30.87	0.89
Fat (g/day)	58.94 ± 43.08	59.36 ± 24.49	0.47
Carbohydrate (g/day)	381 ± 242	363 ± 159	0.92
Dietary fiber (g/day)	29.10 ± 15.54	30.18 ± 13.94	0.61
Thiamin (mg/day)	2.29 ± 1.19	2.21 ± 0.95	0.59
Riboflavin (mg/day)	2.32 ± 0.95	2.40 ± 0.90	0.60
Niacin (mg/day)	21.27 ± 4.03	21.06 ± 7.55	0.90
Pantothenic acid (mg/day)	7.43 ± 2.62	7.85 ± 2.91	0.33
Pyridoxin (mg/day)	2.90 ± 3.45	2.54 ± 1.05	0.16
Folate (μg/day)	515 ± 228	550 ± 182	0.22
Cobalamin (μg/day)	4.49 ± 2.42	4.57 ± 2.67	0.83
Biotin(μg/day)	23.75 ± 9.82	24.08 ± 14.52	0.87
Fe (mg/day)	20.48 ± 11.80	19.93 ± 6.95	0.65
Ca (mg/day)	1308 ± 555	1346 ± 583	0.66
Zn (mg/day)	12.28 ± 6.44	12.30 ± 4.40	0.97
Magnesium (mg/day)	449 ± 305	438 ± 144	0.68
Tryptophan (mg/day)	724 ± 349	729 ± 259	0.90

All values are mean ± SD; energy intake is adjusted for age and sex, all other values are adjusted for age, sex and energy intake. ^a^Sarcopenia was defined based on European Working Group on Sarcopenia in Older People (EWGSOP) definition^27^. ^‡^ANOVA for all variables. *BCAAs* branched-chain amino acids.

General characteristics of study population across tertiles of total and individual BCAAs are summarized Table 3. Patients in the top tertile of leucine intake were more likely to be younger than those in the lowest tertile (P = 0.01). Moreover, participants in the third tertile of isoleucine intake were more physically active than those in the first tertile (P = 0.03). No other significant differences were found comparing extreme tertiles of total and individual BCAAs intake.

**Table 3:** General characteristics of study participants across tertile categories of BCAAs.

	BCAAs		*P*^†^	Valine		*P*^†^	Leucine		*P*^†^	Isoleucine		*P*^†^
	T_1_ (n = 105)	T_3_ (n = 96)		T_1_ (n = 106)	T_3_ (n = 95)		T_1_ (n = 105)	T_3_ (n = 96)		T_1_ (n = 105)	T_3_ (n = 97)	
Age (year)	67.04 ± 7.76	65.42 ± 7.64	0.08	66.75 ± 7.76	66.55 ± 7.72	0.08	66.77 ± 7.63	65.21 ± 7.64	0.01	67.38 ± 8.02	65.36 ± 7.79	0.08
BMI (kg/m^2^)	27.55 ± 3.89	27.40 ± 4.33	0.81	27.65 ± 4.30	27.65 ± 4.32	0.28	27.49 ± 3.87	27.55 ± 4.72	0.72	27.43 ± 3.86	27.64 ± 4.82	0.62
Physical activity (MET-h/w)	17.95 ± 17.05	25.26 ± 25.45	0.09	118.21 ± 17.01	24.80 ± 24.13	0.14	18.15 ± 67.86	25.60 ± 25.38	0.08	17.61 ± 17.11	26.20 ± 25.71	0.03
Female (%)	53.3	44.8	0.33	53.8	48.4	0.74	53.3	44.8	0.33	54.3	46.4	0.51
Alcohol use (%)	11.4	17.7	0.31	11.3	15.8	0.64	11.4	17.7	0.31	10.5	16.5	0.45
Smoking (%)	13.3	14.6	0.62	12.3	11.6	0.85	13.3	14.6	0.62	12.4	12.4	0.97
**Medical history**												
Diabetes (%)	40.3	30.6	0.59	38.7	27.4	0.69	41.9	30.6	0.39	43.5	29	0.27
MI (%)	33.3	25	0.45	30.6	27.8	0.49	30.6	25	0.28	30.6	27.8	0.47
CVA (%)	50	25	0.66	50	12.5	0.47	50	25	0.66	62.5	25	0.23
Asthma (%)	33.3	33.3	0.99	16.7	50	0.53	33.3	33.3	0.99	33.3	50	0.58
Arthritis (%)	20	60	0.40	20	40	0.76	20	60	0.40	20	60	0.41
**Drug history**												
Sexual hormone use (%)	1.9	3.1	0.66	1.9	3.2	0.66	1.9	3.1	0.66	1.9	3.1	0.66
Statin use (%)	40	31.3	0.39	39.6	32.6	0.58	39	31.3	0.40	40	32	0.47
Corticosteroid use (%)	2.9	4.2	0.38	2.8	4.2	0.38	2.9	4.2	0.38	2.9	4.1	0.40

All values are mean ± SD, unless indicated; ^†^ANOVA for continuous variables and Chi-squared test for categorical variables.

*BCAAs* branched-chain amino acids, *BMI* Body mass index.

Dietary and nutrient intakes of study population across tertiles categories of exposure variables are displayed in Table 4. Compared to those in the bottom tertile, individuals in the top tertile of both total and individual BCAAs intake had significantly higher intakes of energy, carbohydrates, proteins, fats, dietary fiber, thiamin, riboflavin, niacin, pantothenic acid, pyridoxin, folate, B12, biotin, iron, calcium, magnesium, zinc and tryptophan (P < 0.001 for all).

**Table 4:** Dietary intakes of study participants across categories of BCAAs, valine, leucine and isoleucine intake.

Variables	BCAAs intake		*P*^†^	Valine intake		*P*^†^	Leucine intake		*P*^†^	Isoleucine intake		*P*^*†*^
	T_1_ (n = 105)	T_3_ (n = 96)		T_1_ (n = 106)	T_3_ (n = 95)		T_1_ (n = 105)	T_3_ (n = 96)		T_1_ (n = 105)	T_3_ (n = 97)	
Energy (kcal/day)	1722. ± 388	2915 ± 1236	< 0.001	1718 ± 387	2889 ± 1250	< 0.001	1726 ± 394	2906 ± 1238	< 0.001	1717 ± 386	2838 ± 1111	< 0.001
Protein (g/day)	59.93 ± 10.21	117.33 ± 35.75	< 0.001	60.41 ± 10.91	117.08 ± 36.26	< 0.001	60.42 ± 11.50	117.20 ± 35.80	< 0.001	59.80 ± 10.09	116.51 ± 35.79	< 0.001
Fat (g/day)	45.38 ± 13.90	75.91 ± 30.56	< 0.001	45.20 ± 13.95	74.02 ± 30.51	< 0.001	45.20 ± 13.94	75.72 ± 30.72	< 0.001	45.19 ± 13.89	75.53 ± 30.76	< 0.001
Carbohydrate (g/day)	284 ± 78.95	465 ± 255	< 0.001	283 ± 78.78	463 ± 258	< 0.001	285 ± 79.74	483 ± 255	< 0.001	283 ± 78.74	445 ± 202	< 0.001
Dietary fiber (g/day)	23.49 ± 7.12	37.32 ± 19.32	< 0.001	23.62 ± 7.36	37.31 ± 19.83	< 0.001	23.71 ± 7.44	37.44 ± 19.33	< 0.001	23.53 ± 7.10	35.99 ± 13.43	< 0.001
Thiamin (mg/day)	1.67 ± 0.45	2.86 ± 1.24	< 0.001	1.67 ± 0.45	2.85 ± 1.26	< 0.001	1.68 ± 0.46	2.85 ± 1.24	< 0.001	1.66 ± 0.45	2.75 ± 0.98	< 0.001
Riboflavin (mg/day)	1.65 ± 0.29	3.25 ± 0.98	< 0.001	1.66 ± 0.29	3.27 ± 0.98	< 0.001	1.67 ± 0.36	3.24 ± 0.98	< 0.001	1.66 ± 0.29	3.18 ± 0.90	< 0.001
Niacin (mg/day)	16.38 ± 3.98	26.46 ± 9.63	< 0.001	16.38 ± 4.03	26.15 ± 9.79	< 0.001	16.49 ± 4.07	26.37 ± 9.64	< 0.001	16.27 ± 3.99	26.20 ± 9.40	< 0.001
Pantothenic acid (mg/day)	5.59 ± 1.15	10.38 ± 3.14	< 0.001	5.59 ± 1.15	10.45 ± 3.13	< 0.001	5.65 ± 1.28	10.35 ± 3.15	< 0.001	5.60 ± 1.15	10.27 ± 3.20	< 0.001
Pyridoxin (mg/day)	1.91 ± 0.50	3.22 ± 1.50	< 0.001	1.90 ± 0.50	3.21 ± 1.53	< 0.001	1.92 ± 0.53	3.21 ± 1.50	< 0.001	1.9 ± 0.5	3.1 ± 1.1	< 0.001
Folate (μg/day)	421 ± 104	673 ± 206	< 0.001	421 ± 106	671 ± 209	< 0.001	425 ± 110	672 ± 206	< 0.001	419 ± 104	663 ± 210	< 0.001
Cobalamin (μg/day)	2.80 ± 0.93	6.85 ± 3.25	< 0.001	2.80 ± 0.95	6.91 ± 3.24	< 0.001	2.81 ± 0.97	6.83 ± 3.27	< 0.001	2.8 ± 0.9	6.8 ± 3.2	< 0.001
Biotin (μg/day)	17.21 ± 4.83	32.15 ± 19.84	< 0.001	17.38 ± 4.99	32.40 ± 19.97	< 0.001	17.40 ± 5.21	32.15 ± 19.84	< 0.001	17.14 ± 4.77	30.10 ± 8.79	< 0.001
Fe (mg/day)	15.53 ± 3.79	25.27 ± 9.99	< 0.001	15.55 ± 3.91	25.04 ± 10.19	< 0.001	15.55 ± 3.82	25.32 ± 9.99	< 0.001	15.47 ± 3.80	25.00 ± 9.83	< 0.001
Ca (mg/day)	893 ± 197	1854 ± 626	< 0.001	891 ± 200	1883 ± 620	< 0.001	907 ± 248	1849 ± 628	< 0.001	902 ± 202	1823 ± 642	< 0.001
Zn (mg/day)	8.70 ± 1.54	16.53 ± 4.96	< 0.001	8.71 ± 1.60	16.51 ± 5.00	< 0.001	8.78 ± 1.74	16.49 ± 4.97	< 0.001	8.69 ± 1.53	16.39 ± 5.02	< 0.001
Magnesium (mg/day)	322 ± 73.16	568 ± 187	< 0.001	324 ± 76.27	565 ± 189	< 0.001	324 ± 77.17	568 ± 187	< 0.001	322 ± 72.77	560 ± 185	< 0.001
Tryptophan (mg/day)	497 ± 85.11	1009 ± 297	< 0.001	504.69 ± 97.50	1002 ± 305	< 0.001	500 ± 92.25	1012 ± 294	< 0.001	494 ± 80.66	1014 ± 290	< 0.001

All values are mean ± SD; ^†^ANOVA for all variables.

**Figure 2:**
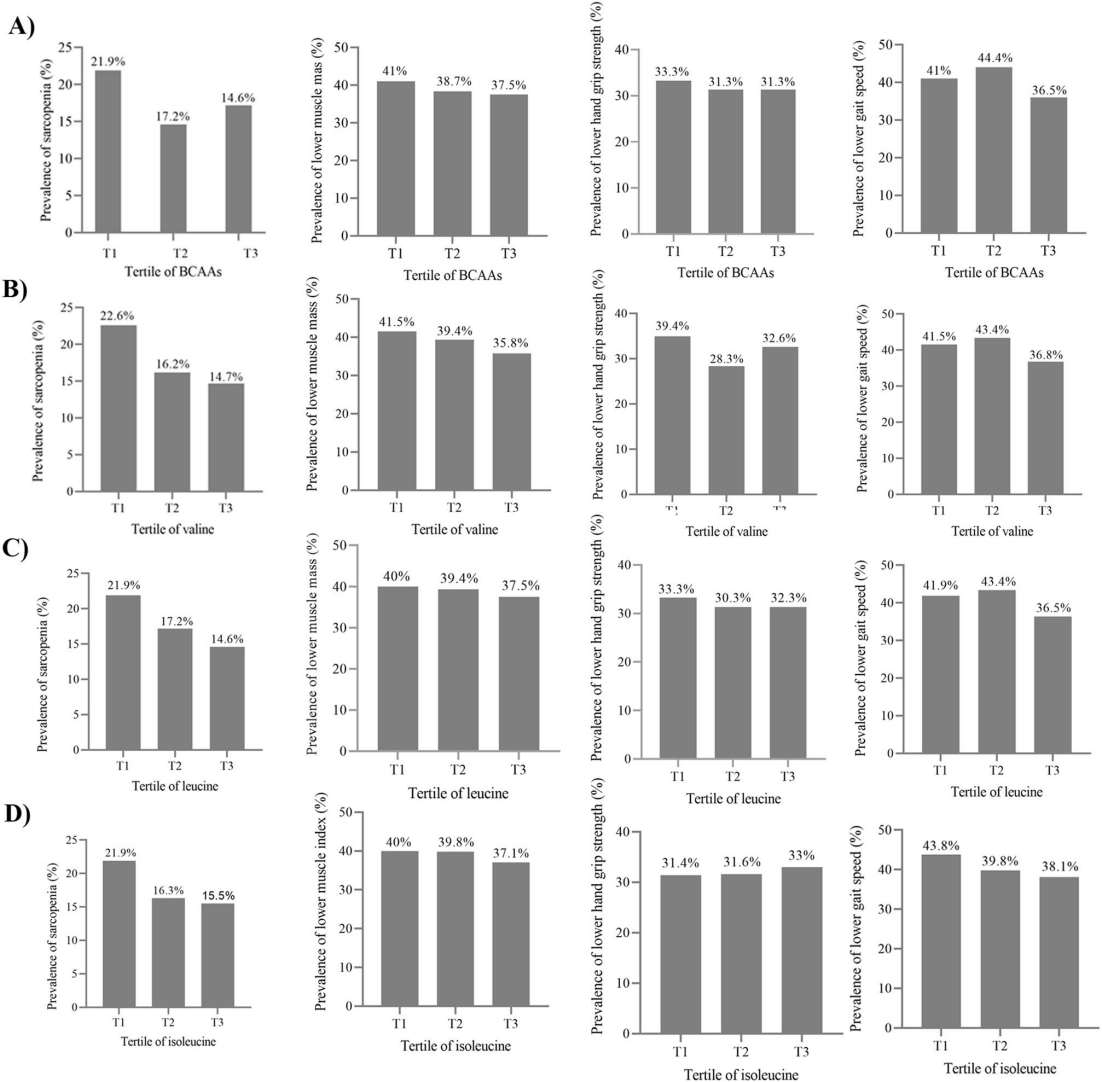
The prevalence of sarcopenia and its components across tertile categories of BCAAS, valine, leucin and isoleucine intakes. A) The prevalence of sarcopenia and its components across tertile categories of BCAAS intake, B) The prevalence of sarcopenia and its components across tertile categories of valine intakes, C) The prevalence of sarcopenia and its components across tertile categories of leucin intake, D) The prevalence of sarcopenia and its components across tertile categories isoleucine intake.

The prevalence of sarcopenia and its components across tertile categories of BCAAS, valine, leucin and isoleucine intakes are showed in Fig. 2. Prevalence of sarcopenia and its components were not significantly different across tertile categories of total and individual BCAAs intake.

Comparing means of muscle mass (kg), hand grip strength (psi), and gait speed (m/s) across tertile categories of total and individual BCAAs, we failed to find any significant differences (Table 5).

**Table 5:** Crude and adjusted means of components of sarcopenia across tertiles of total and individual BCAAs intake.

Variables	BCAAs intake		*P*^†^	Valine intake		*P*^†^	Leucine intake		*P*^†^	Isoleucine intake		*P*^†^
	T_1_ (n = 105)	T_3_ (n = 96)		T_1_ (n = 106)	T_3_ (n = 95)		T_1_ (n = 105)	T_3_ (n = 96)		T_1_ (n = 106)	T_3_ (n = 95)	
**Muscle mass [ASM/h**^**2**^**] (kg)**												
Crude	6.53 ± 0.09	6.70 ± 0.09	0.48	6.56 ± 0.10	6.67 ± 0.09	0.72	6.53 ± 0.09	6.73 ± 0.10	0.32	6.54 ± 0.09	6.71 ± 0.09	0.44
Model^a^	6.56 ± 0.08	6.62 ± 0.09	0.77	6.58 ± 0.08	6.63 ± 0.09	0.94	6.55 ± 0.08	6.65 ± 0.09	0.73	6.58 ± 0.08	6.64 ± 0.09	0.88
**Hand grip strength (psi)**												
Crude	10.79 ± 0.34	11.78 ± 0.38	0.04	10.74 ± 0.34	11.48 ± 0.38	0.31	10.80 ± 0.34	11.7 ± 0.39	0.05	10.77 ± 0.34	11.65 ± 3.84	0.12
Model^a^	10.84 ± 0.24	11.39 ± 0.26	0.30	10.79 ± 0.24	11.28 ± 0.26	0.44	10.83 ± 0.24	11.34 ± 0.26	0.42	10.95 ± 0.24	11.29 ± 0.26	0.51
**Gait speed (m/s)**												
Crude	0.84 ± 0.02	0.86 ± 0.02	0.31	0.84 ± 0.21	0.85 ± 0.21	0.81	0.84 ± 0.02	0.86 ± 0.02	0.38	0.82 ± 0.02	0.86 ± 0.02	0.53
Model^a^	0.82 ± 0.02	0.84 ± 0.02	0.77	0.85 ± 0.02	0.83 ± 0.02	0.88	0.84 ± 0.02	0.84 ± 0.02	0.96	0.83 ± 0.02	0.84 ± 0.02	0.95

Obtained from ANOVA (P < 0.05 significant).

Model^a^: Adjusted for energy, age and sex.

Findings from linear regression analysis of the association between BCAAs and components of sarcopenia revealed no significant association between BCAAs intake and components of sarcopenia (data not shown).

Crude and multivariable-adjusted odds ratios (ORs) and 95% confidence intervals (95% CIs) for sarcopenia and its components by tertiles of BCAAs intake are indicated in Table 6. In the crude model, no significant associations were seen between total BCAAs intake and odds of sarcopenia (OR for comparison of extreme tertiles 0.48, 95% CI 0.19–1.19, P-trend = 0.10) and its components (For muscle mass 0.83, 95% CI 0.39–1.77, P-trend = 0.63; for hand grip strength 0.81, 95% CI 0.37–1.75, P-trend: 0.59; for gait speed 1.22, 95% CI 0.58–2.57, P-trend = 0.56). After adjusting for some potential confounders including age, sex and energy intake, this non-significant relationship did not alter. Further adjustment for other potential confounders did not affect the findings.

**Table 6:** Crude and multivariable-adjusted ORs and 95% CIs for sarcopenia and its components across tertiles of BCAAs intake.

	Tertiles of BCAAs intake			*P trend*
	T_1_	T_2_	T_3_	
**Sarcopenia**				
n (case)	105	99	96	
Crude	1	0.73(0.36–1.48)	0.60(0.29–1.26)	0.17
Model 1^a^	1	0.65(0.31–1.35)	0.47(0.19–1.13)	0.08
Model 2^b^	1	0.66(0.31–1.38)	0.48(0.19–1.19)	0.10
**Lower muscle mass**^**c**^				
Crude	1	0.89(0.51–1.57)	0.86(0.49–1.52)	0.61
Model 1^a^	1	1.21(0.58–2.50)	1.05(0.548–2.06)	0.59
Model 2^b^	1	0.90(0.47–1.70)	0.83(0.39–1.77)	0.63
**Lower hand grip strength**^**d**^				
Crude	1	0.91(0.50–1.64)	0.90(0.50–1.64)	0.74
Model 1^a^	1	0.88(0.46–1.68)	0.88(0.41–1.87)	0.73
Model 2^b^	1	0.89(0.45–1.75)	0.81(0.37–1.75)	0.59
**Lower gait speed**^**e**^				
Crude	1	1.15(0.66–2.0)	0.82(0.46–1.46)	0.53
Model 1^a^	1	1.14(0.62–1.14)	1.06(0.52–2.14)	0.84
Model 2^b^	1	1.23(0.6–2.30)	1.22(0.58–2.57)	0.56

Data are OR (95% CI).

^a^Model 1: Adjusted for age, sex and energy intake. ^b^Model 2: Further adjusted for physical activity, smoking, alcohol consumption, medication use (statin, corticosteroid, estrogen, testosterone), and positive history of disease. ^c^Muscle mass lower than 5.5 (kg/m^2^) for women and 7.0 (kg/m^2^) for men^27^. ^d^Lower muscle strength was defined according previous study^32^. ^e^Gait speeds equal or slower than 0.8 m/s^27^.

Crude and multivariable-adjusted ORs and 95% CIs for sarcopenia and its components by tertiles of valine, leucine and isoleucine intake are shown in Table 7. Similar to total BCAAs intake, we did not find any significant association between individual BCAAs intake and odds of sarcopenia or its components. This was the case when all potential confounders were taken into account.

**Table 7:** Crude and multivariable-adjusted ORs and 95% CIs for sarcopenia and its components across categories of valine, leucine and isoleucine intake.

	Tertile of valine			*P*^†^	Tertile of leucine			*P*^†^	Tertile of isoleucine			*P*^†^
	T_1_	T_2_	T_3_		T_1_	T_2_	T_3_		T_1_	T_2_	T_3_	
n (case)	105	99	96		106	99	95		105	99	96	
**Sarcopenia**												
Crude	1	0.65(0.32–1.32)	0.59(0.28–1.22)	0.14	1	0.73(0.36–1.48)	0.60(0.29–1.26)	0.17	1	0.69(0.34–1.41)	0.65(0.31–1.33)	0.23
Model 1^†^	1	0.55(0.26–1.16)	0.46(0.19–1.09)	0.06	1	0.63(0.30–1.32)	0.47(0.19–1.13)	0.08	1	1.82(0.79–4.18)	1.12(0.50–2.52)	0.14
Model 2^‡^	1	0.55(0.26–1.17)	0.46(0.18–1.13)	0.07	1	0.63(0.30–1.33)	0.48(0.19–1.18)	0.09	1	0.61(0.28–1.31)	0.57(0.24–1.34)	0.18
**Lower muscle mass**												
Crude	1	0.91(0.52–1.60)	0.78(0.44–1.38)	0.40	1	0.97(0.55–1.70)	0.90(0.51–1.58)	0.71	1	0.99(0.56–1.74)	0.88(0.50–1.56)	0.67
Model 1^†^	1	0.81(0.43–1.51)	0.72(0.35–1.49)	0.38	1	0.93(0.50–1.74)	0.86(0.42–1.77)	0.68	1	0.96(0.51–1.80)	0.87(0.43–1.76)	0.70
Model 2^‡^	1	1.31(0.62–2.75)	1.04(0.52–2.05)	0.46	1	0.96(0.51–1.83)	0.89(0.42–1.87)	0.76	1	0.96(0.50–1.83)	0.91(0.43–1.88)	0.80
**Lower hand grip strength**												
Crude	1	0.73(0.40–1.33)	0.90(0.50–1.62)	0.70	1	0.87(0.48–1.56)	0.95(0.52–1.72)	0.86	1	1.00(0.55–1.82)	1.07(0.59–1.93)	0.81
Model 1^†^	1	0.75(0.39–1.44)	0.84(0.40–1.76)	0.62	1	0.86(0.45–1.66)	0.95(0.45–2.00)	0.88	1	1.03(0.53–1.99)	1.09(0.52–2.28)	0.80
Model 2^‡^	1	0.77(0.39–1.52)	0.77(0.36–1.66)	0.50	1	0.87(0.44–1.70)	0.88(0.41–1.89)	0.73	1	0.95(0.44–2.05)	1.02(0.51–2.04)	0.90
**Lower gait speed**												
Crude	1	1.08(0.62–1.88)	0.82(0.46–1.45)	0.51	1	1.06(0.61–1.85)	0.79(0.45–1.40)	0.44	1	0.84(0.48–1.48)	0.79(0.45–1.38)	0.41
Model 1^†^	1	1.06(0.58–1.94)	0.97(0.48–1.95)	0.95	1	0.99(0.54–1.80)	0.99(0.49–2.01)	0.99	1	0.87(0.47–1.60)	1.01(0.51–2.01)	0.97
Model 2^‡^	1	1.14(0.61–2.13)	1.09(0.52–2.27)	0.79	1	1.03(0.55–1.92)	1.15(0.55–2.40)	0.71	1	0.94(0.50–1.77)	1.21(0.59–2.49)	0.61

Data are OR (95% CI).

^†^Model 1: Adjusted for age, sex and energy intake. ^‡^Model 2: Further adjusted for physical activity, smoking, alcohol consumption, medication use (statin, corticosteroid, estrogen, testosterone), and positive history of disease. ^†^Muscle mass lower than 5.5 (kg/m^2^) for women and 7.0 (kg/m^2^) for men^27^. ^‡^Lower muscle strength was defined according previous study^32^. ^§^Gait speeds equal or slower than 0.8 m/s^27^.

## Discussion

In this cross-sectional study among 300 adults including 150 men and 150 women, we failed to find any significant association between dietary intakes of total and individual BCAAs and odds of sarcopenia. The results remained non-significant even after controlling for several potential confounders. To our knowledge, the current investigation is the first reporting the association between dietary intakes of BCAAs and risk of sarcopenia.

In recent decades, sarcopenia, defined as the gradual decline in skeletal muscle mass and function with age, has become a global public health issue as the population ages^34–36^. The prevalence of sarcopenia in developing countries such as the Middle East is higher than that in developed countries^7, 37^, which may be due to the differences in dietary patterns and other non-genetic factors^24, 38^. Numerous studies have explored the role of diet in the prevention of this condition^39–41^. Most have highlighted the role of protein intakes. Proteins can stimulate and regulate muscle protein anabolism, albeit in the presence of BCAAs^15, 42^. In the current study, we failed to find any significant relationship between dietary intakes of total and individual BCAAs and odds of sarcopenia or its components. In a cross-sectional study, plasma concentrations of leucine, isoleucine and valine in sarcopenic and non-sarcopenic individuals were not significantly different^43^. In addition, in the BIOSPHERE study on 68 community dwelling subjects aged 70 and over, individuals with physical disabilities and sarcopenia, as defined by the Foundation for the National Institutes of Health criteria (FNIH), had no significant difference with those who were robust in terms of circulating BCAAs levels^44^. In contrast, in a cross-sectional study, it was found that reduced concentrations of BCAAs were associated with sarcopenia and its components; such that participants in the lowest quartile of BCAA concentrations, whom mean BCAA levels were 453 µmol/L, had a worse sarcopenic indices than those in the highest quartile with a mean serum concentrations of 571 µmol/L^16^. Ottado et al. showed that mean plasma levels of non-fasting BCAAs, valine, leucine and isoleucine in patients with sarcopenia (0.28, 0.16, 0.06 and 0.05 mmol/L, respectively) were lower than those in healthy individuals (0.31, 0.17, 0.07 and 0.06 mmol/L, respectively)^17^. However, they did not consider the protein content of the last meal before blood sampling which might affect their findings^45^. Some studies have shown that there is no significant agreement between dietary intakes and plasma BCAA levels^46^. Others have examined the effect of BCAAs supplementation on muscle health and strength. Hung-Ko et al. conducted a clinical trial to investigate the effect of BCAAs supplementation on 33 sarcopenic and pre-sarcopenic individuals and found that supplementation for five weeks in both groups improved muscle performance including skeletal mass index, gait speed and muscle strength, but these positive effects were lost after a few months^18^. Another study on older malnourished participants reported that a mixture of amino acids including BCAAs for two months improved muscle mass and performance in this population^47^. Overall, based on earlier findings and considering the current study, it seems that BCAAs might have a beneficial effect on muscle strength in a short term but their contribution to sarcopenia and maintaining muscle performance in long term is still questionable. Currently, there is no information about the optimum plasma BCAA levels to prevent sarcopenia. Further studies are needed to examine the optimal plasma levels of BCAA to prevent muscle loss as well as the minimum threshold concentrations of these amino acids required to prevent excessive muscle loss.

Lack of a clear link between dietary intakes of BCAAs and odds of sarcopenia in the current study might be partly explained by the small number of patients with sarcopenia; such that when we categorized study participants based on tertiles of BCAAs intake, we had only 14 individuals with sarcopenia in the highest tertile. Such a small population of sarcopenic patients in the top category resulted in a wide range of confidence intervals forcing the association toward null. Further, in this study, a squeeze bulb dynamometer was used to evaluate and grip strength instead of the gold standard, the Jamar handheld dynamometer^42^, which might have led to misdiagnosis of sarcopenia in some participants.

While we did not find any association between BCAAs intake and sarcopenia, some possible mechanisms may help explaining our hypothesis about existence of the association between BCAAs intake and muscle health and performance. BCAAs especially leucine, have specific positive effects on signaling pathways for muscle protein anabolism by activation of the mammalian target of rapamycin (mTOR) and the downstream phosphorylation of p70S6 kinase (p70S6k) and 4E (eIF4E)-binding protein 1 (4E-BP1) and relevant signaling pathways^18, 48^. BCAAs also decrease muscle protein breakdown by mTOR signaling pathway^49^. However, some studies have shown a potential link between BCAAs and the development of insulin resistance or type 2 diabetes^50–52^. In animal models, decreasing dietary BCAAs resulted in increased energy expenditure and decreased insulin resistance^53^. In fact, BCAAs reduce FEGF-21 by altering fat metabolism and have an adverse effect on insulin sensitivity. Insulin resistance, through the mTor pathway, might affect proteolysis and reduce predominantly oxidative type I fibers that can further decrease glycolytic type II fibers and affect muscle health^53–55^. Therefore, given a positive link between BCAAs and insulin resistance on one hand and the link between insulin resistance and sarcopenia^55^ on the other hand, lack of finding a significant association in the current study might be explained by this mechanism.

The major strengths of the current study are using a reproducible and valid FFQ for assessment of usual dietary intakes, considering several potential confounders in the analysis and being the first study on dietary intakes of BCAAs and sarcopenia. Our investigation had also some limitations. First, lack of data on plasma levels of BCAAs might limit the interpretability of our findings. Second, the sarcopenic individuals in the study might have changed their dietary intakes after observing reduced muscle strength and muscle mass, which the cross-sectional nature of the study did not let us to know these changes. Third, although the dietary evaluation was done by a validated FFQ, these measurements have always measurement errors which might further affect our findings. Finally, due to financial constraints and low access to the only DEXA device available in Tehran (maximum 300 cases), the sample size was small and sampling was limited to the area where the device was located.

In conclusion, we found no significant association between dietary intakes of total and individual BCAAs and odds of sarcopenia and its components after adjusting for potential confounders in this cross-sectional study among Iranian adults. Additional studies, especially with a larger sample size and prospective design, are required to further examine the association between dietary intakes of BCAAs and sarcopenia.

## Abbreviations

BCAAs: Branched-chain amino acids
OR: Odds ratio
CI: Confidence interval
SD: Standard deviation
FFQ: Food Frequency Questionnaire
IPAQ: International Physical Activity Questionnaire
MET-h/week: Metabolic equivalents-hours per week
BMI: Body mass index
SFA: Saturated fatty acid
MUFAs: Mono-unsaturated fatty acids
PUFAs: Polyunsaturated fatty acids
EWGSOP: European Working Group on Sarcopenia
ASM: Appendicular skeletal muscle
PSI: Pound per square inch
mTOR: Mammalian target of rapamycin
